# The future of lab animal facilities in Brazil

**DOI:** 10.1186/1753-6561-8-S4-O28

**Published:** 2014-10-01

**Authors:** Alexandre Viecili

**Affiliations:** 1 Instrulab, Porto Alegre, Rio Grande do Sul, Brazil

## 

The evolution of rodents cages marked major improvements of animal and staff protection. Aspects pertaining to animal welfare and the need for higher flexibility in space used led to the development and success of Individually Ventilated Cages (IVCs), extensively used in modern animal facilities. The concept of "room at cage level" along with the latest innovations in IVCs will be reviewed.

The presentation will highlight how specific abilities to provide native integration among IVCs, laminar flow equipment, washing and decontamination equipment and potentially automation can lead to more efficient planning and later effective exploitation of Lab Animal facilities, as well as to significant bottom.

